# A proposed role for efflux transporters in the pathogenesis of hydrocephalus

**DOI:** 10.3325/cmj.2014.55.366

**Published:** 2014-08

**Authors:** Satish Krishnamurthy, Michael D. Tichenor, Akhila G. Satish, David B. Lehmann

**Affiliations:** 1 Department of Neurosurgery, Upstate Medical University, Syracuse, NY, USA; 2 Upstate Medical University, Syracuse, NY, USA; 3 Department of Engineering, University of Pennsylvania, Philadelphia, PA, USA; 4 Department of Pharmacology, Upstate Medical University, Syracuse, NY, USA

## Abstract

Hydrocephalus is a common brain disorder that is treated only with surgery. The basis for surgical treatment rests on the circulation theory. However, clinical and experimental data to substantiate circulation theory have remained inconclusive. In brain tissue and in the ventricles, we see that osmotic gradients drive water diffusion in water-permeable tissue. As the osmolarity of ventricular CSF increases within the cerebral ventricles, water movement into the ventricles increases and causes hydrocephalus. Macromolecular clearance from the ventricles is a mechanism to establish the normal CSF osmolarity, and therefore ventricular volume. Efflux transporters, (p-glycoprotein), are located along the blood brain barrier and play an important role in the clearance of macromolecules (endobiotics and xenobiotics) from the brain to the blood. There is clinical and experimental data to show that macromolecules are cleared out of the brain in normal and hydrocephalic brains. This article summarizes the existing evidence to support the role of efflux transporters in the pathogenesis of hydrocephalus. The location of p-gp along the pathways of macromolecular clearance and the broad substrate specificity of this abundant transporter to a variety of different macromolecules are reviewed. Involvement of p-gp in the transport of amyloid beta in Alzheimer disease and its relation to normal pressure hydrocephalus is reviewed. Finally, individual variability of p-gp expression might explain the variability in the development of hydrocephalus following intraventricular hemorrhage.

Hydrocephalus is a common brain disorder that affects children and individuals of all ages. It is the most common congenital abnormality in children (one out of 500 births) (1). If left untreated, hydrocephalus can lead to permanent brain damage and result in cognitive and physical handicap.

Contemporary surgical management of hydrocephalus is based on the popular conceptualization of circulation theory. The circulation theory of hydrocephalus states that cerebrospinal fluid (CSF) produced by the choroid plexus flows along specific pathways to be absorbed by the venous sinuses. An obstruction in any part of these pathways leads to hydrocephalus. Surgical management of hydrocephalus is therefore directed at detecting and removing the source of obstruction (such as removal of tumor or blockage of pathways) or diverting the fluid bypassing the obstruction. As such, the most common treatment for hydrocephalus is the surgical implantation of a shunt system to divert the flow of CSF from the ventricles. However, although most cases of hydrocephalus are managed with a shunt system, it is rare for the device to last a lifetime without complications. Treatment of hydrocephalus leads to approximately 38 000 admissions per year in the US. Costs for treatment range from US $1.4-2 billion per year and approximately US $1 billion is spent on the revision of malfunctioning shunts (2).This may be a result of poor shunt design or a flawed approach to treatment.

Circulation theory rests on the assumption that the brain parenchyma is impermeable to water, and is therefore incapable of independently absorbing the CSF that accumulates in the ventricles. However, we have previously seen that the brain is, in fact, permeable to water due to the presence of aquaporin channels and other ion channels (3,4). This permeability of brain parenchyma to water and several other observations question the validity and applicability of circulation theory to design treatment strategies for hydrocephalus.

In brain tissue and in the ventricles, we see that osmotic gradients drive water diffusion in water permeable tissue. Alteration in osmolarity resulting from increase in the concentration of macromolecules and ions has been shown to increase the fluid content and hence the size of the ventricles (5-7). Any osmotic gradient between the ventricular or interstitial CSF and the blood is equilibrated with transport of water between the two compartments. Therefore, water movement into the ventricle is secondary to the presence of osmotic gradients due to excess macromolecules. Thus, water movement into and out of the ventricles is not independent but is dependent upon the presence and resolution of osmotic gradients due to increase or decrease in the macromolecular content (8).

Within this article, we review the role played by osmotic gradients and macromolecular ventricular clearance in hydrocephalus. Macromolecular clearance from the ventricles is a mechanism to establish the normal CSF osmolarity, and therefore ventricular volume. At least two primary pathways of macromolecular ventricular clearance have been studied: paravascular pathways (also known as glymphatic pathways) and olfactory lymphatic pathways.

In particular, we focus on the role played by efflux transporters, specifically p-gp (ABC-B1) in the pathogenesis of hydrocephalus. Efflux transporters are responsible for the transport and clearance of both endogenous (endobiotics) and exogenous (xenobiotics) substances. An understanding of these transporters is critical to designing effective pharmacological treatment for this problematic disorder.

## OSMOTIC GRADIENT AND ITS ROLE IN HYDROCEPHALUS

Previously, hydrocephalus was thought to be a result of an imbalance between CSF production by the choroid plexus and absorption of CSF into the venous sinuses (9,10), Clinical and experimental data to substantiate circulation theory have remained inconclusive (11).

One of the fundamental assumptions of the circulation theory is that the brain parenchyma is impermeable to CSF, and is therefore incapable of absorbing the CSF accumulating within the ventricles. However, the brain parenchyma is permeable to water (12). The molecular basis of this permeability involves specific ion channels that permit water movement with ions as well as aquaporin channels, which permit the free movement of water without changing the ionic environment (3). Aquaporin channels are membrane proteins that have an ion trap and allow movement of water without allowing movement of ions. Several aquaporin channels (aquaporins 1, 4, and 9) are found in the brain tissues (13-15). Aquaporin 4 (AQP4) channels are found in the ependymal cells lining the lateral ventricles and on the end feet of astrocytes. These astrocytes, in particular, contact microvessels in the periventricular white matter and the subpial region of the cerebral cortex (4). The distribution of AQP4 within the brain suggests that water freely moves through the brain parenchyma between the ventricles and vascular system.

As a consequence of water permeability in the brain tissues, osmotic gradients are established by the presence of relatively impermeable, regulated solutes in the ventricles. These gradients result in water transport from the blood plasma into the ventricular CSF (5,6). Driven by osmotic and hydrostatic gradients, water moves between blood plasma and CSF as blood flows through the microvasculature (7,11,16). Therefore, as the osmolarity of ventricular CSF increases within the cerebral ventricles, water movement into the ventricles increases, causing hydrocephalus.

Clinically, high levels of proteins such as thrombopoietin (17), ferritin (18), nerve growth factor (19), chondroitin sulfate proteoglycan (20), transforming growth factor beta 1 (21,22), transforming growth factor beta 2 (20), S-100 protein (23,24), glial fibrillary acidic protein (GFAP) (24), neuron specific enolase (NSE) (24), myelin basic protein (MBP) (24), and vascular endothelial growth factor (25) within ventricular CSF, which may contribute to an elevation in osmolarity, have been observed in patients with hydrocephalus. Additionally, it has been shown that brain edema results from hyponatremia, which is evidence that there is free movement of water from the plasma into the brain tissue (26).

There is substantial experimental evidence relating the osmolarity of the CSF to hydrocephalus. Evidence that increase in CSF osmolarity leads to an increase in ventricular volumes has recently been shown through experiments in our laboratory. Dextran infused into the ventricles of rat brains *in vivo* over 12 days resulted in hydrocephalus, with increasing levels of CSF osmolarity corresponding to proportional increase in ventricle volume (5,6). In other experiments, infusion of proteins FGF-2 (27) and thrombin (28) into the ventricles of animals led to the dilation of ventricles. Other investigators have confirmed the pivotal role that osmotic gradients play in the enlargement of the ventricles and development of hydrocephalus (29-31). A recent study demonstrated significantly faster entry of water from the blood plasma into the CSF with acute increase in CSF osmolarity, which subsequently led to an increase in intracranial pressure (7). These facts suggest that fluid transport from and to the ventricles is secondary to solute or macromolecular transport.

## PATHWAYS FOR CLEARANCE OF MACROMOLECULES

Since osmotic gradients result in hydrocephalus and are caused by the presence of macromolecules in the ventricles, it is particularly important to understand ventricular clearance of macromolecules. We found that macromolecules infused into the ventricles are cleared through the brain parenchyma along the perivascular spaces and along the cribriform plate into the nose (paper being submitted for publication).These findings are consistent with the results of other authors (32-35). Rennels et al infused horseradish peroxidase (HRP) into the lateral ventricles and found that there was a rapid paravascular influx of HRP – a faster influx than can be expected from diffusion (33). The same group found that this transport of HRP through the paravascular pathways was limited by focal cerebral edema (36). Zhang et al injected India ink into cerebral white and gray matter and into the subarachnoid space and found that the tracer was transported along specific paravascular pathways (37). Further, subarachnoid injection resulted in transport of the tracer along the paravascular pathways as well as through the lymphatics in the nose. The observation that macromolecules or tracers in the ventricles are transported along paravascular pathways has been recently confirmed by observing the tracer movement using confocal microscopy (38) and magnetic resonance (MR) imaging (39).These pathways have been referred to as the glymphatic pathways or system (38,39). Zhang et al found that the particulate matter that was injected was rapidly and efficiently ingested by perivascular cells (37). These authors highlighted both the paravascular and the nasal lymphatic pathways in macromolecular clearance and their immunological significance (38).

## LOCATION AND FUNCTION OF EFFLUX TRANSPORTERS

The regulation of transport of macromolecules into and out of the brain is vital to maintaining homeostasis and ensuring a proper environment for neural activity. There are a number of both endobiotic and xenobiotic compounds (Table 1) that have been documented to be cleared from the brain at the blood-brain barrier (BBB). Efflux transporters are located along the blood-brain barrier as well as blood-CSF barrier (BCSFB) and play an important role in maintaining homeostasis of compounds and solutes in the brain milieu as well as the CSF (40). The brain capillary endothelial cells form a tight barrier and are characterized by the absence of any fenestrations (41). These anatomical features of the BBB restrict the paracellular leakage of endogenous or exogenous compounds from the brain into the capillaries (42). As a result, transcellular transport across the BBB and subsequent clearance by these efflux transporters is critical in the elimination of unwanted solutes from the brain (41). The two important classes of active efflux transporters on the BBB are the solute transporter carrier family (SLC) (this family includes organic anion transporting polypeptides [OATP/SLCO] and organic anion transporters [OAT/SLC22A]) and ATP-binding cassette (ABC) transporters, in particular multidrug resistance-associated proteins and p-gp (41).

**Table 1:** Examples of compounds cleared from the brain

Compound	
Endogenous	Exogenous/xenobiotic
Amyloid-β (28)	3′-azido-3′-deoxythymidine (107)
Cholecystokinin-A (47)	2',3′-dideoxyinosine (108)
Cholecystokinin-B (47)	Vinca Alkaloids (108)
Neuropeptide Y (88)	Doxorubicin (109)
Albumin (89)	Cyclosporin A (109)
Transferrin (89)	Digoxin (109)
Iron (90)	Methotrexate (109)
Dehydroepiandrosterone sulfate (91)	Pemetrexed (110)
Estrone-3-sulfate (92)	Prednisolone (110)
Creatinine (93)	Morphine (111)
GABA (94)	Dextran (83)
Leptin (95)	Amprenavir (112)
Guanidinoacetate (96)	Indinavir (113)
Taurine (97)^1^	Verapamil (114)
L-Proline (98)	Phenobarbital (115)
Glycine (99)	Lamotrigine (116)
Taurocholic acid (99)	Felbamate (116)
Prostaglandin D2 (100)	Dantrolene (116)
L-Aspartic Acid (101)	Digoxin (117)
L-Glutamic Acid (102)	Rhodamine-123 (117)
Indoxyl Sulfate (102)	Nortriptyline (118)
Hippuric Acid (103)	
3-carboxy-4-methyl-5-propyl-2-furanpropanoic Acid (103)	
Quinolinic Acid (103)	
5-hydroxyindole Acetic Acid (103)	
Homovanillic Acid (103)	
3-hydroxyglutaric (103)	
Methylmalonic Acid (104)	
17beta-estradiol-D-17beta-glucuronide (104)	
D-serine (105)	
Hypoxanthine (106)	
Adenine (107)	
Adenosine (107)	

Members of the ABC transporter family, which include p-gp, MRP 1-6, and BRCP, have been shown to eliminate an abundance of macromolecular substrates from the brain including anticancer drugs, immunosuppression drugs, corticoids, analgesics, HIV protease inhibitors, cytokines, antidepressants, antibiotics, and diagnostic dyes, such as Rhodamine-123, just to name a few (43). Barbiturates, many of which are p-gp substrates (44), injected into the CSF compartments of dogs are rapidly cleared into the bloodstream (45). Cholecystokinin-A (CCK-A) and CCK-B have both been shown to be p-gp substrates (46). One study found that intraventricular injection of CCK-A led to less feeding in rats, presumably being cleared into the bloodstream and interacting with peripheral receptors (47).

Xenobiotics and other endobiotics are recognized by xenobiotic sensors also known as adopted “orphan” nuclear receptors (pregnane X receptor [PXR] and constitutive androstane receptor [CAR]) in the cytoplasm (48). Both nuclear receptors can be activated by endogenous ligands and a wide range of xenobiotics (49), which include a broad array of prescription drugs, herbal remedies, steroid hormones, bile salts, and vitamins (50). These nuclear receptors can then up-regulate the functional expression of p-gp (51). Inhibition of PXR and CAR activities could reduce p-gp expression, whereas activation of PXR and CAR can induce p-gp expression (50).

P-gp expression on the plasma membranes of the brain capillary endothelial cells (on the luminal or blood-side of the endothelial cell), pericytes, and astrocytes show that p-gp is expressed in all cells that make up the BBB (52).The paravascular pathways may clear macromolecules through the BBB, particularly by using efflux transporters located on the BBB (38). Specifically, p-gp is of great interest in this role due to the fact that it is the most abundant efflux transporter on the BBB and has broad substrate specificity (53). In addition, p-gp transporters are distributed along the pathways of the macromolecular clearance explained above, along the microvessels in the thalamus, hippocampus, cerebellar, and cerebral cortex from 22 weeks of gestation to adulthood (54). Therefore, we are going to focus the discussion on the role played by efflux transporters with a special emphasis on p-gp.

## P-GLYCOPROTEIN AND ITS ROLE IN THE PATHOPHYSIOLOGY OF HYDROCEPHALUS

We hypothesize that impaired macromolecular clearance out of the brain will result in increased concentration of endobiotics or xenobiotics in the brain interstitium and ventricular fluid. This change will result in water influx into the ventricles resulting in hydrocephalus. Optimal efflux transport and clearance of macromolecules prevents the development of hydrocephalus. Down-regulation or inhibition of the efflux transport will result in hydrocephalus, while up-regulation of the efflux transport will relieve hydrocephalus. We will examine the current evidence that supports this concept.

Inhibition and induction of p-gp has been shown to affect the amount of substrates that remain within the brain or enter it. Induction of p-gp at the BBB has been shown to decrease the concentration of such substrates in the brain extracellular fluid (55-59). Conversely, inhibition of p-gp on the BBB has been shown to increase substrate concentrations in the brain (60-64). Several studies point to the possible role of p-gp in diseases of the central nervous system including Alzheimer disease (65,66), Parkinson disease (65,66), Huntington’s disease (66), Creutzfeldt-Jakob’s disease (66), amyotrophic lateral sclerosis (66), and epilepsy (66). The role that p-gp plays in numerous neurodegenerative diseases is believed to be in part due to the protection that it provides at the BBB to maintain homeostasis in the brain and protect it against toxic compounds. Its function along the BBB is believed to prevent the accumulation of potentially harmful compounds in the brain by actively removing them into the peripheral circulation (67).

The importance of macromolecular clearance in maintaining normal neurological function is exemplified by the pathophysiology of Alzheimer disease. Normally amyloid-β is cleared from the brain via efflux transporters on the BBB, particularly by p-gp and MRP1 (68). When amyloid-β is insufficiently cleared from the brain, toxic Aβ oligomeric and aggregated species accumulate in the brain, which results in Alzheimer disease (69). Manipulation of these efflux transporters can lead to either increased or decreased clearance of Aβ from the brain. Rifampicin and caffeine, which are two compounds that up-regulate p-gp activity, were shown to increase the clearance of Aβ from the brain (70). On the other hand, knockout of two efflux transporters p-gp and BCRP increased the amount of Aβ within the brain (71).

There is a considerable overlap of Alzheimer disease and normal pressure hydrocephalus, with some authors reporting nearly 50% of patients with probable NPH having evidence of AD (72). Several studies have shown that these two diseases co-exist, and the presence of amyloid plaques in the brain tissues of patients with normal pressure hydrocephalus is indicative of poor prognosis for successful treatment of hydrocephalus (71-75).Recently, positron emission tomography (PET) has been used to detect the presence of amyloid plaques in patients clinically suspected to have normal pressure hydrocephalus (76).

Macromolecules are transported out of the ventricles and the brain into the serum even in the presence of hydrocephalus. Clinical studies show that there are several endobiotics that are cleared from CSF into the serum and these include S-100b, GFAP, NSE, and MBP. Vascular endothelial growth factor (VEGF) is elevated in CSF of patients with hydrocephalus (77). VEGF was increased several fold in the CSF in experimental chronic hydrocephalus and the increase was significantly correlated with ventricular volume (78). Excess VEGF in the CSF is known to decrease p-gp activity, thus contributing to hydrocephalus (79).

Experimental evidence in models of congenital hydrocephalus further supports the role played by p-gp in the pathogenesis. P-gp expression in the subcortical white matter and periventricular tissues (paravascular pathways) of H-Tx rats was absent when compared to normal animals (80). In another study (81), H-Tx rats were also found to have reduced nasal lymphatic clearance of CSF, suggesting that the paravascular/lymphatic pathways were impaired. In support of our experimental model of hydrocephalus, dextrans are known to be substrates of p-gp and they are distributed along both the olfactory pathways as well as the paravascular pathways (82).

One of the most common causes of hydrocephalus is intraventricular hemorrhage (IVH). IVH is a cause of significant morbidity and mortality as a complication of neonatal germinal matrix hemorrhage and adult intracerebral hemorrhage (83). IVH causes hydrocephalus in 25–50% of these individuals and results in additional damage to the brain (83). Despite the fact that the amount of blood in the ventricles plays a role in the development of hydrocephalus, it is not known why some patients develop hydrocephalus and some do not. The role played by p-gp as an efflux transporter can potentially explain this variability.

There is considerable variation of expression of p-gp in general population. P-gp is the gene product of MDR1 (ABCB1). The MDR1 gene is subject to single nucleotide polymorphisms, which is associated with decreased function of p-gp between individuals (84). A well-known example of this variability affecting function is in relation to the opiate analgesics.

Mu opioid receptor agonists have identical target receptor affinities adjusted for dose (85). Despite this characteristic, individual opioids exhibit a marked variation in their ability to induce clinically-relevant analgesia despite equipotent mu opioid receptor doses administered systemically, a characteristic that is explained entirely on the basis of the affinity of p-gp for an individual opioid (analgesia is inversely proportional to the affinity of the p-gp efflux transporter for the substrate drug) (86). Hence, loperamide, a potent mu opioid receptor agonist, does not produce either euphoria or analgesia, being marketed over-the-counter in the USA as an antidiarrheal preparation, due to its local inhibition of gut motility (88). Evidence that loperamide has *in vivo* potent mu opioid agonist properties in the brain and that this effect is solely due to p-gp efflux has been clearly supported by pretreatment with a potent p-gp inhibitor prior to loperamide administration (87).

It is still unclear why some patients with IVH develop hydrocephalus and others do not. One of the possible explanations is that there are genetic differences between individuals in how well they clear the blood and the blood products from the ventricles. Based on the discussions above, we hypothesize that if a patient has a genetic variant with decreased functionality of p-gp, then the potential for eliminating macromolecules from the ventricles will be reduced in the presence of IVH, resulting in hydrocephalus.

In conclusion, hydrocephalus results from abnormal osmotic gradients that favor water transport into the ventricles. These abnormal gradients are due to a change in the macromolecular and ionic content in the ventricular CSF. Efflux transporters, in particular p-gp, play a key part in the clearance of macromolecules, both endogenous and exogenous, out of the ventricles and the brain, thus maintaining the milieu interior. We have summarized existing literature with reference to p-gp and provided a context for focusing on this transporter with regards to hydrocephalus. However the details regarding the exact role played in hydrocephalus are yet to be clarified.
